# Aurora kinase and FGFR3 inhibition results in significant apoptosis in molecular subgroups of multiple myeloma

**DOI:** 10.18632/oncotarget.26180

**Published:** 2018-10-02

**Authors:** Utkarsh Painuly, Vijay Ramakrishnan, Teresa Kimlinger, Linda Wellik, Jessica Haug, Wilson Gonsalves, Lintao Bi, Zhongxia Huang, S. Vincent Rajkumar, Shaji Kumar

**Affiliations:** ^1^ Division of Hematology, Mayo Clinic, Rochester, MN, USA; ^2^ 4th Department of Internal Medicine–Hematology University Hospital Hradec Kralove and Charles University in Prague, Faculty of Medicine in Hradec Kralove, Hradec Králové, Czech Republic; ^3^ The Department of Hematology and Oncology, China-Japan Union Hospital attached to JiLin University, Changchun, Jilin, China

**Keywords:** multiple myeloma, FGFR3, Aurora kinase, apoptosis, proliferation

## Abstract

Aberrant expression of proteins involved in cell division is a constant feature in multiple myeloma (MM), especially in high-risk disease. Increasingly, therapy of myeloma is moving towards individualization based on underlying genetic abnormalities. Aurora kinases are important mediators of cell cycle and are up regulated in MM. Functional loss of Aurora kinases results in genetic instability and dysregulated division leading to cellular aneuploidy and growth arrest. We investigated the role of Aurora kinase inhibition in MM, using a small molecule inhibitor A1014907. Low nanomolar A1014907 concentrations induced aneuploidy in MM cell lines independent of underlying cytogenetic abnormalities by inhibiting Aurora Kinases. However, A1014907 induced more pronounced and dose dependent apoptosis in cell lines with t(4;14) translocation. Translocation t(4;14) is observed in about 15% of patients with MM leading to constitutive activation of FGFR3 in two-thirds of these patients. Further investigation of the mechanism of action of A1014907 revealed potent FGFR3 pathway inhibition only in the sensitive cell lines. Thus, our results show that aurora kinase inhibition causes cell cycle arrest and aneuploidy with minimal apoptosis whereas inhibiting both aurora kinase and FGFR3 activity induced potent apoptosis in MM cells. These results support clinical evaluation of A1014907 in MM patients with t(4;14) translocation and/or FGFR3 expression.

## INTRODUCTION

Aurora kinases (A, B and C) are highly conserved serine threonine kinases whose functions mediate different stages of cell division [1]. Aurora A is localized to the centrosomes and spindle poles during mitosis and facilitates centrosome maturation, spindle assembly and chromosome separation [2–4]. Aurora B is localized to the centromere till metaphase and subsequently relocates to the spindle midbody through telophase and cytokinesis [5]. Aurora B along with survivin, borealin, and INCENP is a member of the chromosome passenger complex. This complex functions in several important steps in mitosis including chromosome condensation, spindle assembly checkpoint control and cytokinesis [6–8]. The function of Aurora kinase C, is relatively less understood in mitosis. It is thought to function in the absence of Aurora kinase B [9]. Depletion of Aurora-A leads to mitotic arrest followed by the induction of polyploidy in cells lacking p53 [10]. Low levels of Aurora A contributes to decreased activity of Cyclin B/CDK1, a G2M regulatory protein complex, further contributing to cell cycle arrest [11, 12]. During cell cycle progression, abnormally formed spindles are detected at mitotic checkpoint, which then arrests the cells in mitosis. Depletion of Aurora-B eliminates activation of mitotic checkpoint, leading to formation of tetraploid cells. As mitotic checkpoint remains inactivated, these cells fail to arrest at mitosis [13, 14].

Increased expression of Aurora kinases have been observed in various tumors including multiple myeloma (MM) [15, 16]. The lack of expression of the aurora kinases in non-dividing cells and the increased expression in cancer cells makes this an attractive therapeutic target [15]. Aurora kinase as a therapeutic target in MM has been explored with several inhibitors in pre clinical setting, including ENMD-2076, MLN8237, AZD1152, AT9283 and VE-465 [17–21]. Out of these, AT9283 and VE-465 are the only inhibitors that target both Aurora A and B kinases. None of these inhibitors are currently under clinical evaluation in MM.

We studied the role of Aurora A and B kinase inhibition in a heterogeneous group of MM cell lines using a small molecular inhibitor of Aurora Kinase A and B, A1014907. A1014907 induced G2M arrest and polyploidy in all MM cell lines indicating Aurora Kinase A and B inhibition. In particular, we observed increased sensitivity of cell lines with t(4;14) translocation to A1014907. Upon further studies, we present clear evidence that in addition to aurora kinases, A1014907 inhibits FGFR3, a receptor overexpressed primarily in MM cells with t(4;14) translocation.

## RESULTS

### Cell lines with t(4;14) translocation show increased sensitivity to A1014907

**Figure 1:**
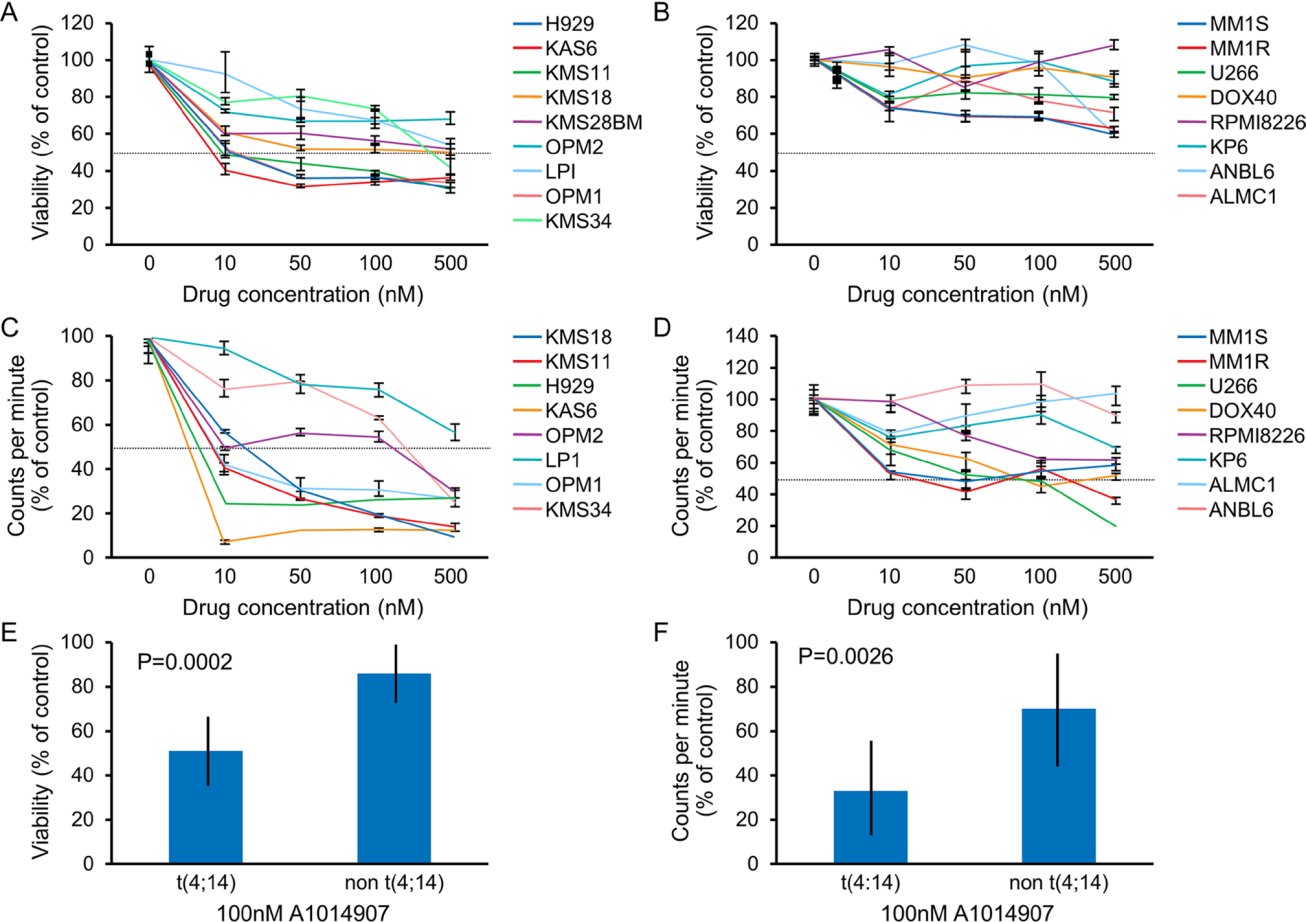
When MM cell lines were incubated with indicated doses of A1014907 for 72 hrs, we observed potent dose dependent decrease in (**A**) viability and (**C**) proliferation in t(4;14) MM cell lines and relatively less decrease in (**B**) viability and (**D**) proliferation in non t(4;14) MM cell lines. (**E**) Mean % viability and (**F**) Mean % proliferation of all t(4;14) cell lines and all non t(4;14) cell lines after treatment with 100 nM of A1014907 for 72 hrs shows significantly more cell death and growth arrest respectively in the t(4;14) cell lines. *P* values show statistical significance.

We first assessed the cytotoxic effects of A1014907 on MM cells. For this, we treated a panel of MM cell lines with various doses of A1014907 for 72 hrs and examined the cytotoxicity induced by the drug. We observed that cell lines OPM2, KMS11, KMS18, KMS28BM, H929, KAS6, LP1, OPM1 and KMS34 were significantly more sensitive to A1014907 treatment when compared to the other cell lines examined (Figure 1A, 1B and 1E). The IC50 values are shown in Table 1. Similar differential effects were also seen when we performed thymidine incorporation assays to examine the anti-proliferative effects induced by A1014907 (Figure 1C, 1D and 1F). The IC50 values observed in proliferation assay are shown in Table 2. The cell lines sensitive to A1014907 in both assays, namely, KMS11, KMS18, KMS28BM, H929, KAS6, LP1, OPM1 and KMS34 all have t(4;14) translocation and express FGFR3. Thus at lower doses A1014907 is able to inhibit proliferation in both t(4;14) as well as non t(4;14) cells suggestive of aurora kinase inhibition in both these groups of cell lines. Out of the t(4;14) cell lines, KMS28BM, KMS34, H929 and KAS6 all express wild type FGFR3 and KMS11 (Y373C), KMS18 (G382D) and OPM1 and OPM2 (K560E) have activating mutations in FGFR3. LP1 has a non activating mutation in FGFR3 (F384L).

**Table 1:** IC50 values of A1014907 on both the t(4;14) and non t(4;14) cell lines used in this study

t(4;14) translocation and FGFR3 mutational status	IC50	Non t(4;14) translocation	IC50
Kas6 (wt FGFR3)	10 nM	MM1S	>500 nM
KMS11 (Y373C)	50 nM	MM1R	>500 nM
KMS18 (G382D)	500 nM	DOX40	>1000 nM
KMS28BM (wt FGFR3)	500 nM	RPMI8226	>1000 nM
H929 (wt FGFR3)	10 nM	U266	>1000 nM
OPM2 (K560E)	>500 nM	KP6	>500 nM
KMS34 (wt FGFR3)	500 nM	ALMC1	>500 nM
LPI (F384L)	>500 nM	ANBL6	>500 nM
OPM1 (K560E)	50 nM		

Results show enhanced cytotoxicity of A1014907 on cell lines with t(4;14) translocation.

**Table 2:** IC50 values of A1014907 on both the t(4;14) and non t(4;14) cell lines used in this study as determined by thymidine incorporation assays

t(4;14) translocation and FGFR3 mutational status	IC50	Non t(4;14) translocation	IC50
Kas6 (wt FGFR3)	10 nM	MM1S	50 nM
KMS11 (Y373C)	10 nM	MM1R	50 nM
KMS18 (G382D)	50 nM	DOX40	100 nM
KMS28BM (wt FGFR3)	50 nM	RPMI8226	>500 nM
H929 (wt FGFR3)	10 nM	U266	50 nM
OPM2 (K560E)	500 nM	KP6	>500 nM
KMS34 (wt FGFR3)	500 nM	ALMC1	>500 nM
LPI (F384L)	>500 nM	ANBL6	>500 nM
OPM1 (K560E)	10 nM		

The IC50 values as determined by this assay were not very different between the two groups of cell lines though t(4;14) cells did show enhanced sensitivity to A1014907.

### A1014907 is effective in the presence of tumor promoting bone marrow stromal cells

**Figure 2:**
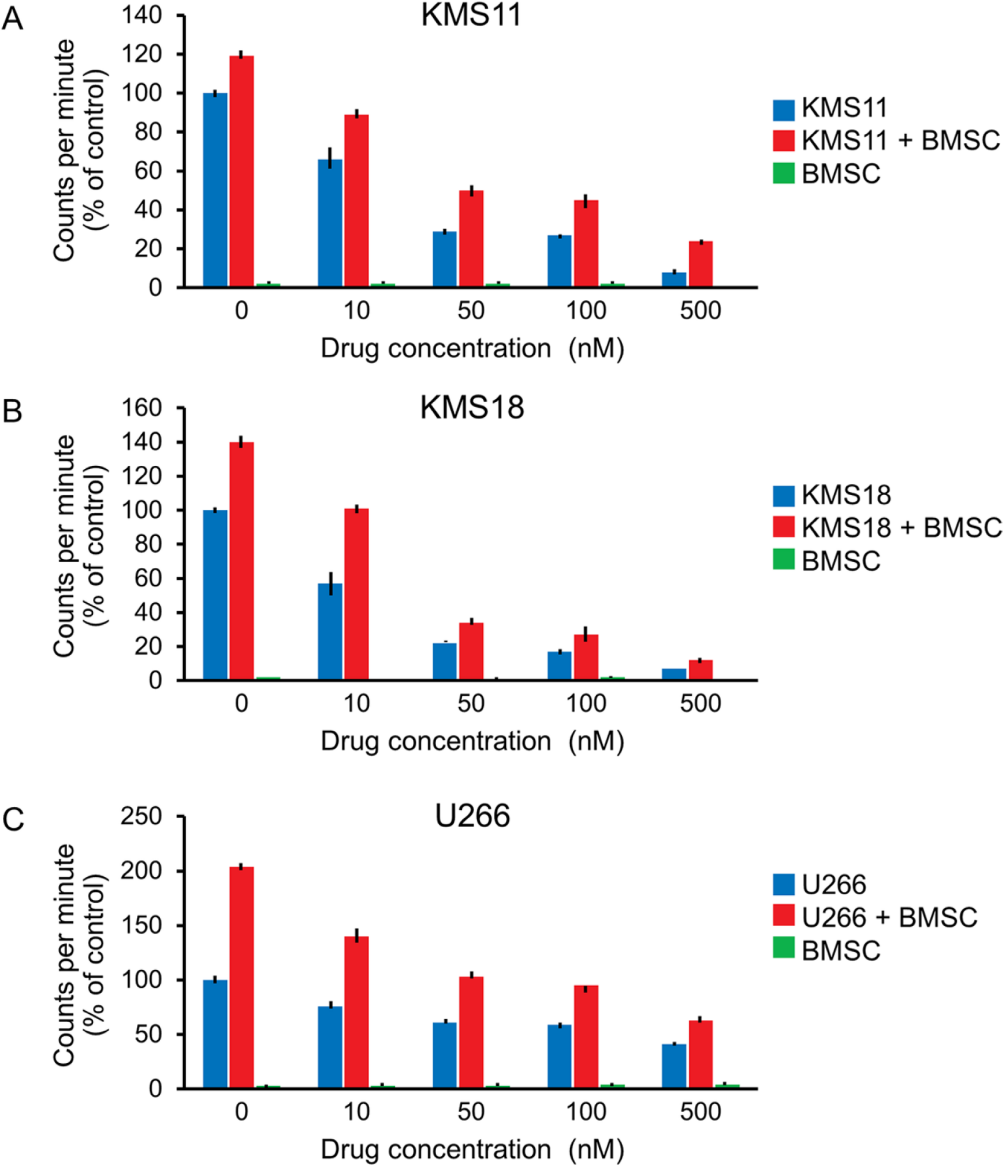
t(4;14) MM cell lines KMS11 and KMS18 and non t(4;14) MM cell line U266 were co-cultured with BMSCs derived from a MM patient followed by treatment with indicated doses of A1014907 As shown in (**A**–**C**), A1014907 was able to overcome the tumor protective effects of the BMSCs and inhibit the proliferation of MM cells even in the presence of BMSCs.

Bone marrow stromal cells (BMSCs) are important cellular components of the tumor microenvironment, whose interaction with MM cells contribute to disease progression and resistance to existing therapies [22]. We therefore examined if A1014907 was able to overcome the protective effects of BMSCs. We observed that A1014907 was able to inhibit proliferation of MM cells even in the presence of BMSCs in both t(4;14) (Figure 2A and 2B) and non t(4;14) cells (Figure 2C).

### A1014907 induces polyploidy in all MM cell lines and pronounced apoptotic cell death in t(4;14) MM cell lines

**Figure 3:**
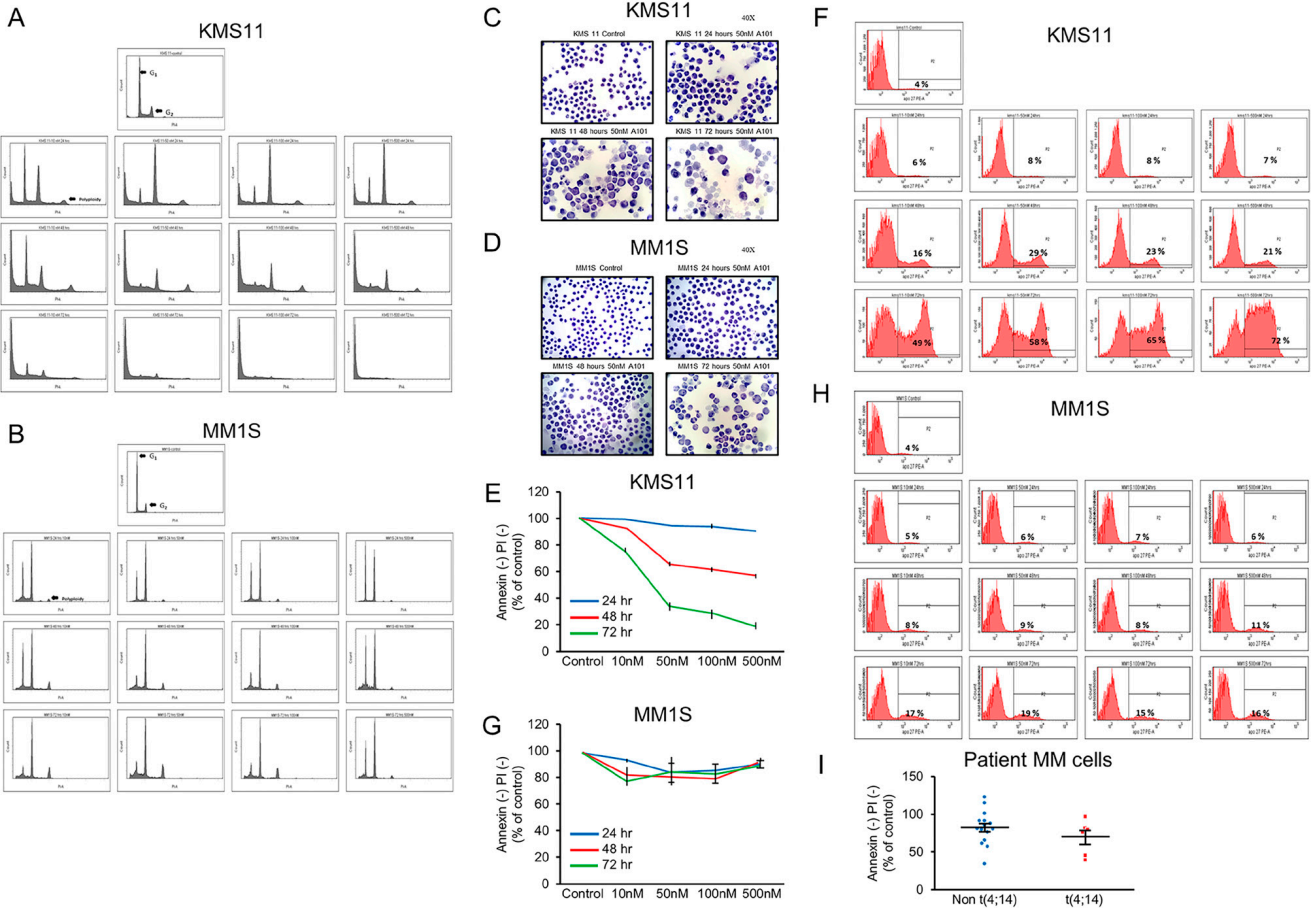
When (**A** and **C**) a t(4;14) MM cell line KMS11 and (**B** and **D**) a non t(4;14) MM cell line MM1S were treated with indicated doses of A1014907, we observed G2M arrest followed by polyploidy in both cell lines. In (A) KMS11, we observed G2M arrest at 24 hrs and polyploidy at 24 and 48 hrs and increased apoptosis at 72 hrs. In (B) MM1S, we observed prolonged G2M arrest and polyploidy at all time points with very little apoptosis. In (C and D) we observed that A1014907 caused an increase in cell size with varying nuclear numbers and sizes typically seen during defects in cell division in both KMS11 and MM1S cells. Pictures were taken at 40× magnification. When (**E** and **F**) KMS11 and (**G** and **H**) MM1S were treated with indicated doses of A1014907, we observed profound apoptosis in KMS11 cells as evidenced by (E) annexin/PI staining and (F) Apo 2.7 staining. This increase in annexin/PI and Apo 2.7 positivity was not observed in MM1S cells (G and H respectively). (**I**). We treated plasma cells from 15 patients without t(4;14) translocation and 6 MM patients with t(4;14) translocation with 1000 nM of A1014907 for 72 hrs. Following this, annexin/PI staining was performed to examine apoptosis induced by the drug.

We next assessed if A1014907 induced cell division aberrations in both t(4;14) as well as non t(4;14) cells. For this experiment, we used KMS11, a t(4;14) and MM1S, a non t(4;14) cell line. G2M arrest and polyploidy was observed in both cell lines. This was evident as early as 24 hours with 10 nM A1014907 treatment. (Figure 3A and 3B). To confirm that the appearance of the 8N peak was due to the ability of A1014907 to induce polyploidy and not because of the drug’s ability to cause G2/M arrest, we treated MM1S cells with TG101209, a Jak2 inhibitor that we had shown in an earlier study to induce G2/M arrest. Results showed that TG101209 induced G2/M arrest without causing polyploidy further confirming that A1014907 does indeed induce polyploidy (Supplementary Figure 1). Morphological analysis showed that untreated control cells appeared to be of uniform cell and nuclear size (Figure 3C and 3D). However, A1014907 caused the cells to increase in size with varied nuclear numbers/sizes within each cell indicative of cell division abnormality and possibly aurora kinase inhibition. (Figure 3C and 3D). Given that A1014907 induced more potent cell death in cells with t(4;14) translocation than in cells lacking this translocation, we next performed assays to confirm if the cell death occurred through induction of apoptosis. For this, we treated KMS11 and MM1S cells with indicated concentrations of A1014907 for various time points. We observed that A1014907 induced potent apoptotic cell death in KMS11 cells (Figure 3E and 3F). However, MM1S and U266 cells showed resistance to A1014907 treatment with minimal increase in apoptosis (Figure 3G, 3H and Supplementary Figure 2).

### A1014907 shows enhanced activity against patient cells with t(4;14) translocation

We then examined apoptosis induced by A1014907 on MM patient cells with or without the t(4;14) translocation. For this, we treated non t(4;14) (*n =* 15) and patients with t(4;14) translocation (*n =* 6) with A1014907 for 72 hrs. We then measured apoptosis by annexin/PI staining. Our results showed a trend towards enhanced activity of A1014907 on patients with t(4;14) translocation (Figure 3I) though the difference was statistically insignificant due to small patient numbers. The patient disease characteristics are included in Table 3.

**Table 3:** The patients used in this study and their disease characteristics

Patient #	Disease stage	Disease State	No. of prior therapies	Prior ASCT	Cytogenetics	t(4;14)	Para protein	BMPC%	Labelling Index	Viability (% of control at 1 μM)
1	MGUS	no treatment	No	No	Trisomies	No	IgG kappa	5	1.4	123.1
2	SMM	no treatment	No	No	Trisomies	No	IgG kappa	30	0.2	65.8
3	SMM	no treatment	No	No	Del13, t(4;14), +1q	Yes	IgG lambda	20	1.2	62.2
4	SMM	no treatment	No	No	t(11;14)	No	IgG kappa	30	0.2	77.5
5	SMM	no treatment	No	No	Del13, trisomies	No	IgG kappa	10	0.3	91.8
6	MM	Newly diagnosed	None	No	Trisomies	No	IgG kappa	30	0.9	115.8
7	MM	Newly diagnosed	None	No	Normal	No	IgG kappa	20	0.1	82.4
8	MM	Newly diagnosed	None	No	Trisomies	No	IgG kappa	90	1.1	88.6
9	MM	Newly diagnosed	None	No	Normal	No	IgA lambda	5	2.2	80.6
10	Relapsed MM	Active relapse	4	Yes	Del13, Del17p, trisomies	No	IgG kappa	30	10.5	34.1
11	Relapsed MM	Active relapse	2	Yes	Trisomies	No	IgG lambda	20		91.3
12	MM	Newly diagnosed	None	No	Trisomies	No	IgG kappa	30	1.1	81.1
13	Relapsed MM	Active relapse	4	Yes	Del13, Del14	No	IgG lambda	25	0.6	102.4
14	Relapsed MM	Responding dz	5	Yes	Del13, Del17p, Trisomies	No	IgG kappa	0	N/A	86.8
15	Relapsed MM	Responding dz	9	Yes	del13, t(11;14)	No	Lambda	5	1.2	58.2
16	Relapsed MM	Active relapse	2	No	del13, t(4;14)	Yes	IgA kappa	90	2.1	76.7
17	Relapsed MM	Responding dz	12	Yes	del13, t(4;14)	Yes	IgD lambda	30	2.8	81.8
18	MM	Responding dz	1	No	del13, t(4;14)	Yes	IgG lambda	5	1.4	39.4
19	MM	Responding dz	1	No	del13, t(4;14)	Yes	IgA kappa	10	0.8	44.9
20	Relapsed MM	Responding dz	8	Yes	t(4;14), Trisomies	Yes	IgG lambda	90	1.5	79.2
21	Relapsed MM	Active relapse	3	Yes	del13, t(4;14), Trisomies	Yes	IgG kappa	10	0.8	96.7

### A1014907 inhibits Aurora Kinases A and B and proteins involved in cell cycle machinery

**Figure 4:**
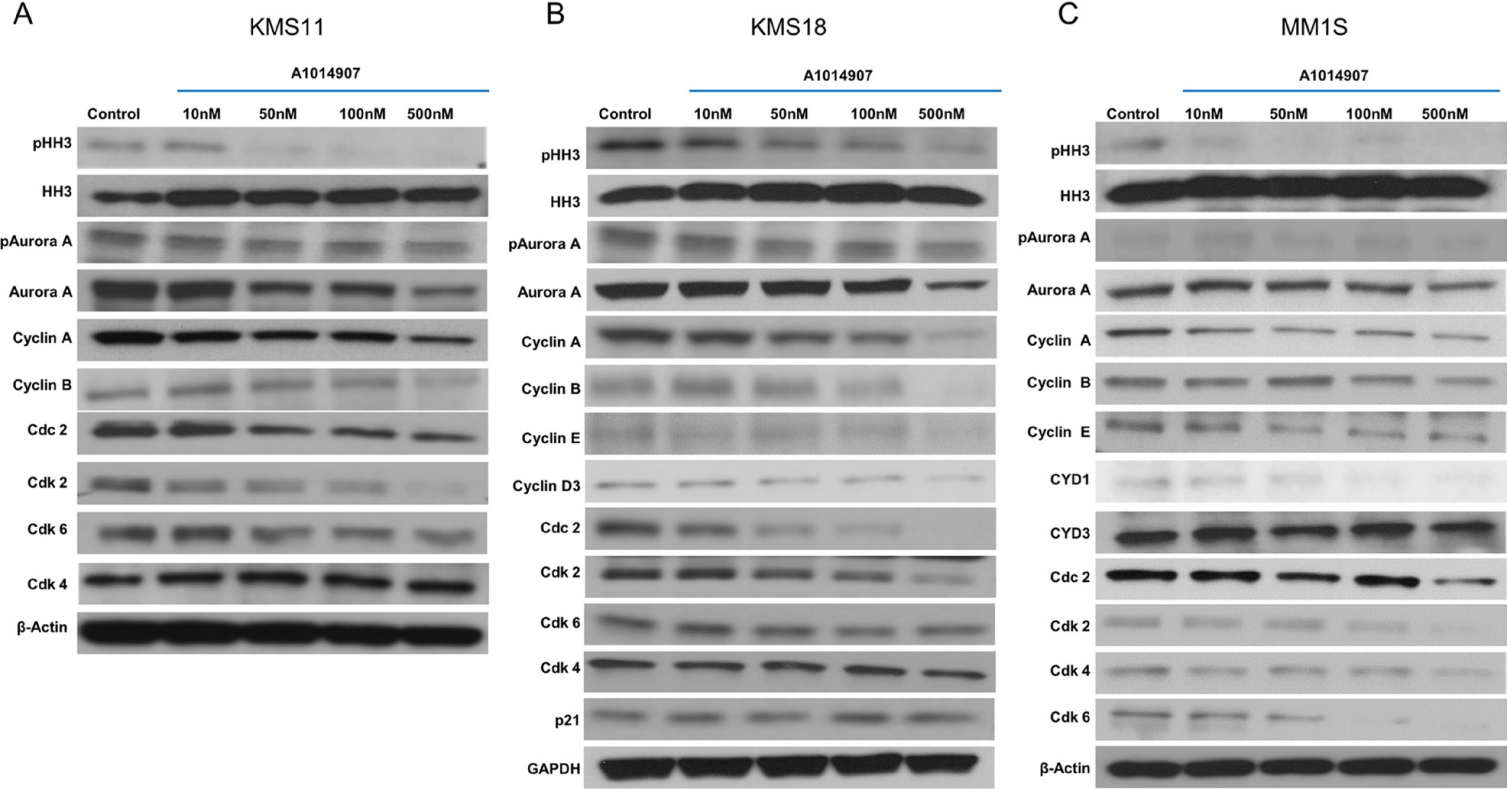
(**A**) KMS11, (**B**) KMS18 and (**C**) MM1S cells were incubated with indicated concentrations of A1014907 for 48 hrs and the expression levels of pHH3, pAuroraA, the various cyclins and cyclin dependent kinases were examined by western blots.

Next, we examined the mechanism of action of A1014907. We treated two t(4;14) cell lines KMS11 and KMS18 and one non t(4;14) cell line MM1S cells with indicated concentrations of A1014907 and examined the expression levels of aurora kinases and proteins involved in cell cycle progression. As expected, we saw down regulation of phospho Aurora A and phospho Histone H3, a substrate of Aurora B and a biomarker of mitosis in all the three cell lines (Figure 4A–4C). Surprisingly, we also observed that A1014907 caused down regulation of total Aurora A. While we do not know the mechanism contributing to this, we speculate that A1014907 acts by blocking aurora A phosphorylation and in addition also by inducing the degradation of Aurora A. Our results from Figure 3A and 3B clearly showed that A1014907 caused accumulation of cells in the G2M stage of the cell cycle in addition to polyploidy. We therefore examined the levels of proteins involved in cell cycle progression. A1014907 treatment reduced levels of Cdc2, cyclins A and B (Figure 4A–4C). Cdc2-Cyclin A complex regulates late S phase and early M phase while Cdc2-Cyclin B complex regulates M phase of the cell cycle. Thus, our data suggests that reduction in the level of Cdc2 and its binding partners cyclins A and B could be major factors contributing to the observed G2M arrest (Figure 3A and 3B). Cdc2-Cyclin B complex is a key regulator of nuclear envelope breakdown [23, 24]. Its inhibition could further contribute to a lack of or abnormal karyokinesis, producing abnormal cells with unequal DNA content (Figure 3C and 3D).

### A1014907 inhibits FGFR3 in t(4;14) cells promoting apoptosis

**Figure 5:**
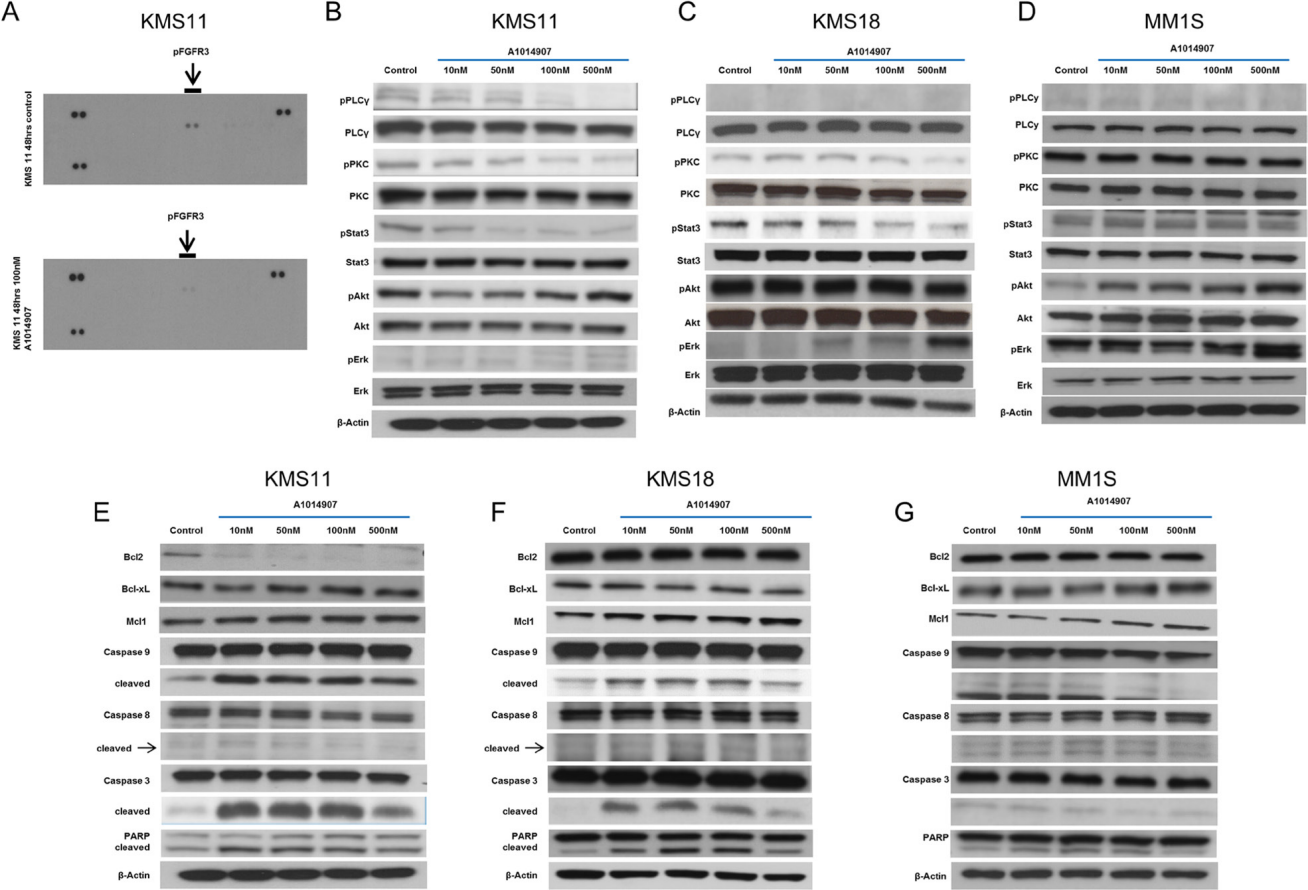
(**A**) KMS11 cells were left untreated (top panel) or treated with 100 nM of A1014907 for 48 hrs. A receptor tyrosine kinase array was then performed and we observed down regulation of pFGFR3 levels post drug treatment. (**B**) KMS11, (**C**) KMS18 and (**D**) MM1S cells were treated with indicated concentrations of A1014907 for 48 hrs and the expression levels of canonical pathway members activated by FGFR3, namely PLCγ, PKC, Stat3, Akt and Erk were examined by western blotting. (**E**) KMS11, (**F**) KMS18 and (**G**) MM1S cells were treated with indicated concentrations of A1014907 for 48 hrs and the expression levels of caspases, PARP, Bcl2, Bcl-Xl and Mcl1 were examined by western blotting.

Our results so far suggest that A1014907 inhibits the proliferation and causes polyploidy in all MM cell lines (Figure 1C and 1D and Figure 3A and 3B) through the down regulation of Auroras A and B. (Figure 4A–4C). However, A1014907 induced significant cell death only in MM cells with t(4;14) translocation (Figure 1A and 1B and Figure 3E–3H), which is associated with increased FGFR3 expression [25]. We therefore hypothesized that A1014907, in addition to being an aurora kinase inhibitor was also able to inhibit FGFR3 causing increased apoptosis in cells with t(4;14) translocation. We performed a receptor tyrosine kinase (RTK) array using KMS11 cells left untreated or treated with indicated dose of A1014907. The results showed down regulation of pFGFR3 by A1014907 (Figure 5A).

Activated FGFR3 can cause the up regulation of various signaling pathways including the PLCγ/PKC, Jak2/Stat3, PI3K/Akt, and Mek/Erk pathways [26]. To better understand which of these signaling pathways are involved in A1014907 induced apoptosis, we performed western blotting using lysates from KMS11, KMS18 and MM1S cells. We observed inhibition of pPLCγ, pPKC and pStat3 in cell lines KMS11 and KMS18, which are both sensitive to A1014907 (Figure 5B and 5C), In MM1S, the cell line lacking FGFR3 expression, we did not observe down regulation of any of these proteins again suggesting specific inhibition of FGFR3 by A1014907 (Figure 5D).

Next, we examined levels of the Bcl2 family of anti apoptotic proteins and observed down regulation of Bcl-2 and Bcl-Xl in KMS11 and KMS18 respectively (Figure 5E and 5F). In addition, we observed activation of the intrinsic apoptotic pathway as shown by increased levels of cleaved caspases 9 and 3 (Figure 5E and 5F) and inactivation of PARP (Figure 5E and 5F) all indicating increase in cell death. It must be noted that such differences in apoptotic proteins were absent in MM1S cells (Figure 5G).

### A1014907 activity is dependent on FGFR3 expression but not t(4;14) translocation

**Figure 6:**
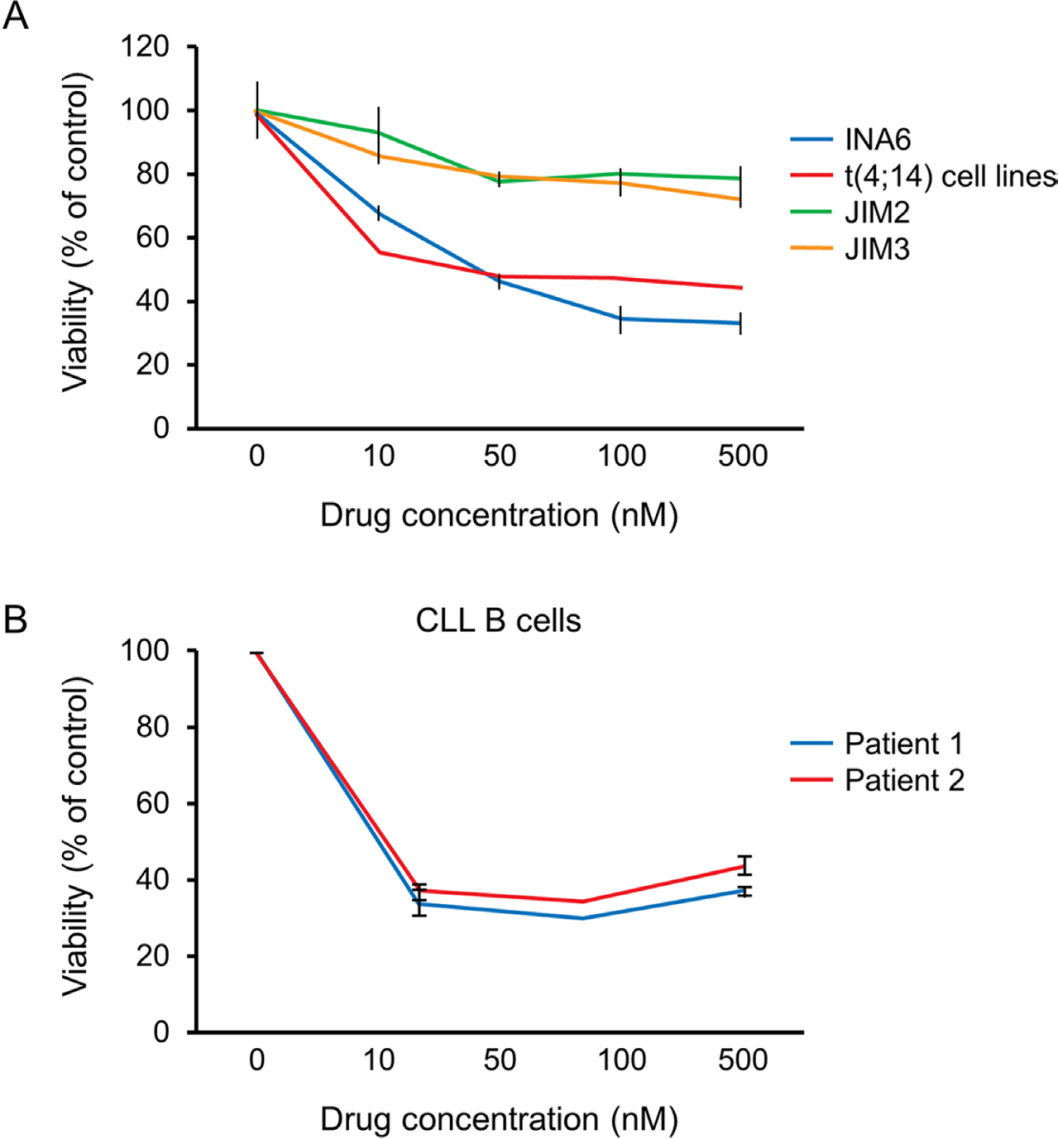
(**A**) INA6, JIM2 and JIM3 were exposed to indicated doses of A1014907. A1014907 induced significantly more cytotoxicity in INA6 when compared to JIM2 and JIM3. The cytotoxicity induced in INA6 was similar to the mean cytotoxicity induced in cell lines with t(4;14) translocation. The mean cytotoxicity was calculated from the results shown in Figure 1A. (**B**) CLL B cells from two patients were treated with indicated doses of A1014907 for 72 hrs. A1014907 induced potent cytotoxicity in both the patient cells similar to our observations on FGFR3 positive MM cell lines.

To further confirm that A1014907 inhibits FGFR3, we checked the sensitivity of INA6, a non t(4;14) line that overexpresses FGFR3 to A1014907. We observed that INA6 was sensitive to A1014907 like the t(4;14) cell lines (Figure 6A). To further confirm that the activity of A1014907 is dependent on FGFR3 expression and not due to the presence of t(4;14) translocation, we examined the effect of A1014907 on two MM cell lines JIM2 and JIM3, both of which are t(4;14) cell lines but negative for FGFR3 expression. A1014907 was unable to induce pronounced cell death in both these cell lines (Figure 6A). Recently, it was shown that Chronic Lymphocytic Leukemia (CLL) B cells express FGFR3, which potentiates their growth and survival [27]. We therefore treated CLL B cells from two patients with indicated doses of A1014907. We observed clear induction of cell death in both these patients (Figure 6B). Both the patients were positive for FGFR3 expression as determined by flow cytometry (data not shown). Taken together, we show that A1014907 induces cell death in FGFR3 expressing MM cells. FGFR3 expression could therefore serve as a biomarker to predict for sensitivity to A1014907.

## DISCUSSION

MM is a genetically heterogeneous disease with about 50% of patients presenting with translocations involving the Immunoglobulin heavy chain (IgH) locus, often called the non hyperdiploid group and the remaining with trisomies of odd numbered chromosomes, often called the hyperdiploid group [28–30]. The common translocations observed in MM patients in the order of decreasing frequency include t(11;14), t(4;14), t(14;16), t(6;14) and t(14;20) [29]. Almost all these patients express high levels of at least one D-type cyclin, thus contributing to cell cycle checkpoint abnormalities [31–33]. In addition, aurora kinases are aberrantly expressed in myeloma further adding to abnormalities in the cell division process [16]. Here, we have examined the preclinical activity of a novel aurora kinase inhibitor A1014907. Our findings show that aurora kinase inhibition by A1014907 causes potent cell cycle arrest and polyploidy across all MM cell lines evaluated. However, aurora kinase inhibition alone appears to be insufficient to induce significant apoptosis. In addition to being an aurora kinase inhibitor, A1014907 is able to potently inhibit FGFR3. Simultaneous inhibition of Aurora kinases along with FGFR3 signaling induces cell cycle arrest, polyploidy and also apoptosis in FGFR3 expressing cells.

t(4;14) is the second most common translocation observed in MM patients with a frequency of 15% [29]. Recently, it has been shown that MM patients presenting with a combination of International Scoring System 3, high lactate dehydrogenase and t(4;14) and/or del (17p) treated with novel agents and autologous stem cell transplantation are at a high risk of progression and death [34]. Therefore, therapies that can overcome the adverse prognosis associated with t(4;14) translocations are urgently required. Prior studies have shown that MM patients and cell lines with t(4;14) have a high centrosome index (CI) and overexpress aurora kinases [16]. We therefore expected to observe increased activity of A1014907 on MM cell lines and patients with t(4;14). However, we observed a dramatic difference in the sensitivity of t(4;14) containing MM cell lines when compared with the non t(4;14) cell lines. This is unlikely to be explained merely by aurora kinase inhibition. Aurora kinase inhibitors have been examined as anti-MM agents in other studies and a few such inhibitors inhibited other receptor tyrosine kinases (RTKs) in addition to aurora kinases [17, 20]. We therefore hypothesized that A1014907 inhibited FGFR3 expressed in about two-thirds of patients with t(4;14) and in several MM cell lines with t(4;14). Our results show that A1014907 inhibits aurora kinases and FGFR3. In cells lacking FGFR3 expression, A1014907 caused cell cycle arrest in majority of them but failed to induce marked apoptosis. In cells expressing wt FGFR3 or those with activating mutations in FGFR3 (Y373C or G382D), A1014907 caused potent cell cycle arrest and in addition pronounced apoptosis. Furthermore, we dissected the FGFR3 mediated down stream pathways and showed clear evidence that FGFR3 inhibition by A1014907 resulted in down regulation of both the PLCγ/PKC and Stat3 signaling pathways leading to apoptosis induction in these cell lines. FGFR3 is also observed in MM independent of the t(4;14) translocation [35, 36] and hence A1014907 could also be effective in those patients.

Taken together, our results clearly show preferential activity of A1014907 on MM cells with FGFR3 expression. t(4;14). Clinical evaluation of A1014907 on relapsed/refractory MM patients with t(4;14) and/or FGFR3 expression is planned.

## MATERIALS AND METHODS

### MM cell lines and patient cells

Cell lines MM1S, MM1R, RPMI8226, OPM2 and U266, were kindly provided by Dr. Jonathan Keats (TGen, Phoenix, AZ). Kas6 was kindly provided by Dr. John Lust (Mayo Clinic, Rochester, MN). KMS11, KMS18, KMS28BM, KMS34, LP1, OPM1, JIM2, JIM3 and INA6 were kindly provided by Dr. Leif Bergsagel (Mayo Clinic, Scottsdale, AZ). H929 was purchased from ATCC (Manassas, VA, USA) and DOX40 was kindly provided by Dr. William Dalton (Moffitt Cancer Center, Tampa, FL, USA). ALMC1, ANBL6 and KP6 were kindly provided by Dr. Diane Jelinek (Mayo Clinic, Rochester, MN). All cell lines except Kas6, KP6, ANBL6, ALMC1 and INA6 were cultured in RPMI 1640 media (Mediatech Inc., Manassas, VA) containing 2 mM L-glutamine (Invitrogen, Grand Island, NY), 100 U/mL penicillin, 100 μg/mL streptomycin and 10% fetal bovine serum (Mediatech, Inc.). Kas6 and INA6 were cultured in same media but in the presence of 4 ng/mL of IL6 and 1 ng/ml IL6 respectively (R&D Systems, Inc., Minneapolis, MN). ALMC1, ANBL6 and KP6 were cultured in IMDM media (Invitrogen) containing 2mM L-glutamine (Invitrogen), 100 U/ml penicillin, 100 μg/mL streptomycin, 10% fetal bovine serum (Mediatech, Inc.) and supplemented with 1 ng/ml IL6 (R&D Systems). Bone marrow aspirates were obtained from MM patients after informed consent under a protocol approved by the Mayo Clinic Institutional Review Board in adherence with the Declaration of Helsinki. Samples were processed to obtain MM cells or bone marrow stromal cells (BMSCs) as detailed elsewhere [37]. CLL B-cells were obtained after informed consent under a protocol approved by the Mayo Clinic Institutional Review Board in adherence with the Declaration of Helsinki. Samples were processed as mentioned elsewhere.

### Drugs

A1014907 was synthesized and provided by Abbott Laboratories Ltd under a Material Transfer Agreement (MTA). Stock solutions were made in DMSO, diluted in RPMI-1640 and subsequently stored at −20° C.

### MTT and proliferation assays

Cytotoxic effects of A1014907 were measured using 3-(4, 5-dimethylthiazol-2-yl)-2, 5-diphenyl tetrasodium bromide (MTT) (Chemicon International Inc., Temecula, CA) colorimetric assay as described earlier [38–40]. Tritiated thmidine uptake assays were utilized to assess the anti-proliferative effects of A1014907 on MM cells when cultured either alone or in combination with BMSCs as described earlier [38–40].

### Morphological analysis

Cells were fixed on glass slides using methanol. Slides were dried and deparaffinized followed by staining with Hematoxylin dye. Stained slides were rinsed, dehydrated using ethanol and xylene and finally mounted with resinous mounting medium. Microscopic images were taken at 40× magnification highlighting morphological changes post A1014907 treatment.

### Cell cycle analysis

Cells were treated with indicated doses of A1014907 for 24, 48 or 72 hrs. Cells were harvested, counted and washed with PBS following which 2 ml of cold 85% ethanol was added to the pellet while the tubes were vortexed. The tubes were left at 4° C overnight. Subsequently, the cells were pelleted and washed twice with PBS. The pellet was then resuspended in 0.1 ml of 5 μg/ml RNase (Sigma-Aldrich, St. Louis, MO) and incubated at 37° C for 30 minutes. PBS (0.9 ml) and 10 μl of 1 mg/ml propidium iodide (PI) (Sigma-Aldrich) were added and samples were held at 4°C till they were run on a Canto flow cytometer (BD Biosciences, San Jose, CA) and analyzed using FlowJo software (Tree Star, Ashland, OR).

### Apoptosis assays

Apoptosis induction by A1014907 in MM cell lines and patient cells was measured by annexin V/PI staining and flow cytometry [38–40]. Briefly, cells were washed twice in Annexin Binding Buffer (ABB) (10 mM HEPES pH 7.4, 140 mM NaCl, 2.5 mM CaCl2). 100 μl cells (10^7^ cells/ml) were stained with 3 μl of annexin V-FITC (Caltag, Burlingame, CA) for 15 minutes at room temperature. Cells were again washed with ABB and re-suspended in 500 μl ABB containing 5 μl of 1 mg/ml PI (Sigma-Aldrich) and run on a Canto flow cytometer (BD Biosciences).

### Western blotting

Cells were lysed in RIPA buffer (50 mM HEPES (pH 7.4), 150 mM NaCl, 1% Triton X-100, 30 mM sodium pyrophosphate, 5 mM EDTA) containing Halt Phosphatase Cocktail (Thermo Fisher Scientific, Rockford, IL), 1 mM phenylmethylsulfonyl-fluoride (PMSF) (Thermo Fischer) and protease inhibitor cocktail (PIC) (Sigma-Aldrich). Protein lysate concentrations were measured using BCA assay (Thermo Fisher). Equal amounts of protein were loaded on Tris-Glycine gels and transferred onto nitrocellulose membranes (Bio-Rad, Hercules, CA). All antibodies were purchased from Cell Signaling Technology (Danvers, MA). Antigen-antibody complexes were detected using enhanced chemiluminescence (GE Healthcare, Piscataway, NJ).

### Receptor tyrosine kinase (RTK) array

Human phosphor-RTK array was obtained from R&D Systems Inc, which allowed simultaneous screening of 49 different phosphorylated RTK’s. The array was processed following the manufacturer’s instructions. Briefly, protein extracts obtained from untreated or drug treated cells were added onto antibody coated nitrocellulose membrane After overnight incubation at 4° C, the membrane was washed to remove excess antibodies and incubated with anti phospho tyrosine HRP Detection antibody for two hours followed by detection of the antigen antibody complex using chemiluminescence.

### FGFR3 expression by flow cytometry

The CLL patient cells were obtained after informed consent and processed and stained for surface FGFR3 expression as described elsewhere [27].

### Statistical analyses

All experiments were performed thrice and the error bars represent one standard deviation. Students *t* test was used to examine significance in Figure 1E and 1F.

## SUPPLEMENTARY MATERIALS FIGURES


