# Supplementary material for: Aurora kinase and FGFR3 inhibition results in significant apoptosis in molecular subgroups of multiple myeloma
Source: Oncotarget. 2018 Oct 2;9(77):34582–94. doi: 10.18632/oncotarget.26180 (PMC6195373; doi:10.18632/oncotarget.26180)
Supplement: Supplementary file 1 [file oncotarget-09-34582-s001.pdf]

Aurora kinase and FGFR3 inhibition results in significant apoptosis in molecular subgroups of multiple myeloma

SUPPLEMENTARY MATERIALS

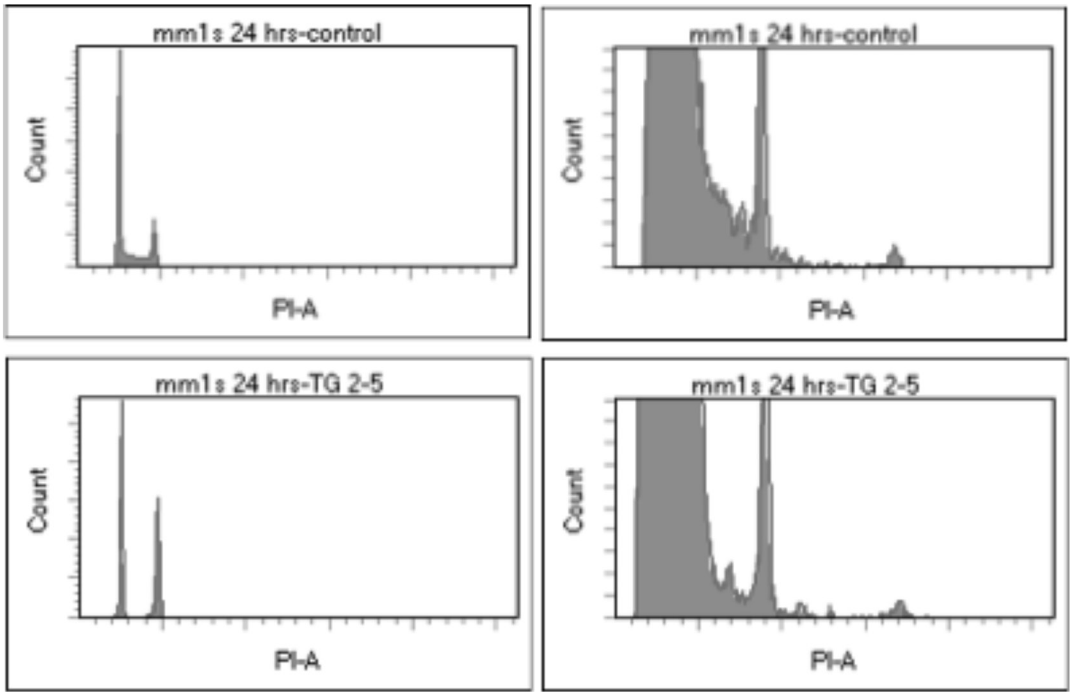

Supplementary Figure 1: We incubated MM1S cells with 2.5 µM of TG101209 for 24 hrs and performed cell cycle analysis. We observed that TG101209 induced G2M arrest (shown in left panel). In order to check if TG101209 induced polyploidy, we compressed the axis to examine the 8N peak and any changes induced by the drug in this population. We observed no increase in the 8N peak suggesting that TG101209 did not induce polyploidy (shown in right panel).

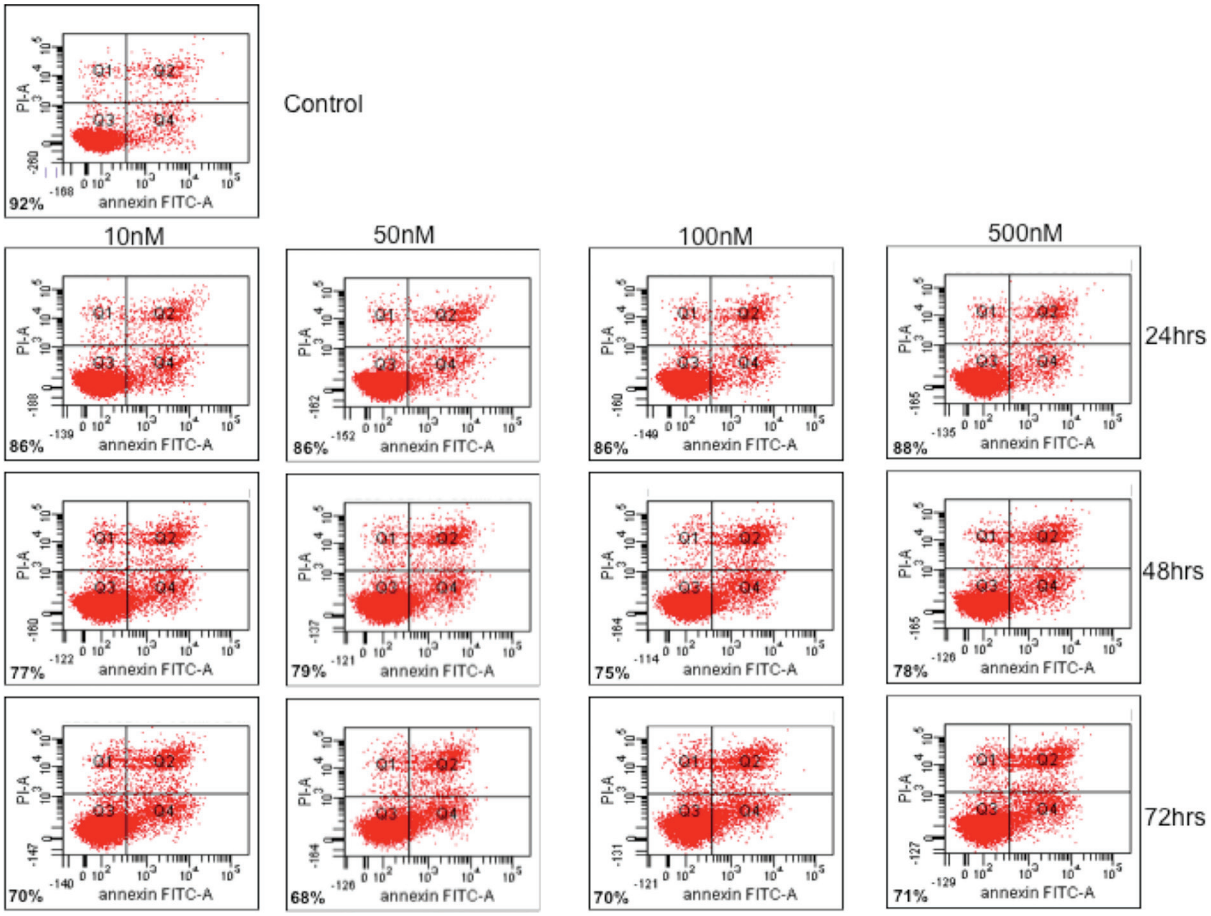

Supplementary Figure 2: We incubated U266 cells with indicated concentrations of A1014907 for 24, 48 or 72 hrs and measured apoptosis by annexin/PI staining. We observed that A1014907 did not induce significant increase in apoptosis. % Viable cells (annexin and PI negative) are indicated in the figure.
